# A simple technique for checking nipple height in breast reduction and mastopexy

**DOI:** 10.1308/rcsann.2024.0042

**Published:** 2024-07-31

**Authors:** YR Chin, D Oliver

**Affiliations:** ^1^ University Hospitals Plymouth NHS Trust, UK; ^2^ Royal Devon University Healthcare NHS Foundation Trust, UK

## Background

Marking the new position of nipple areola complex (NAC) is crucial in the successful outcome of breast reduction (BBR) and mastopexy. Although techniques for BBR and mastopexy may vary, determining the height of the new NAC position is common to all procedures. It is surgically difficult to correct an NAC that has been placed too high.

## Technique

**Figure 1:**
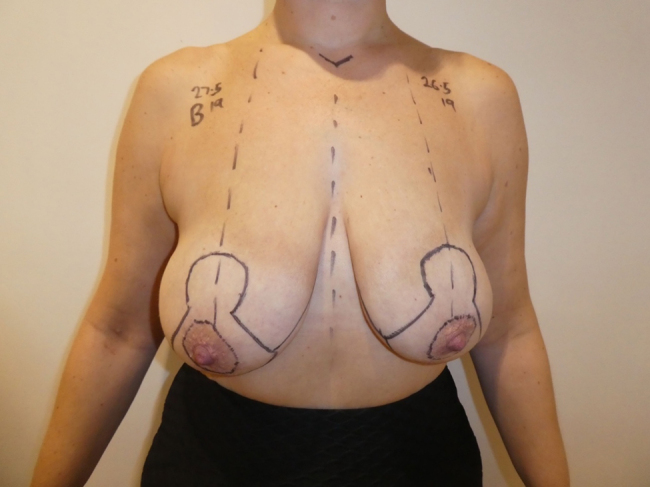
Wise-pattern marking for breast reduction or breast augmentation

**Figure 2:**
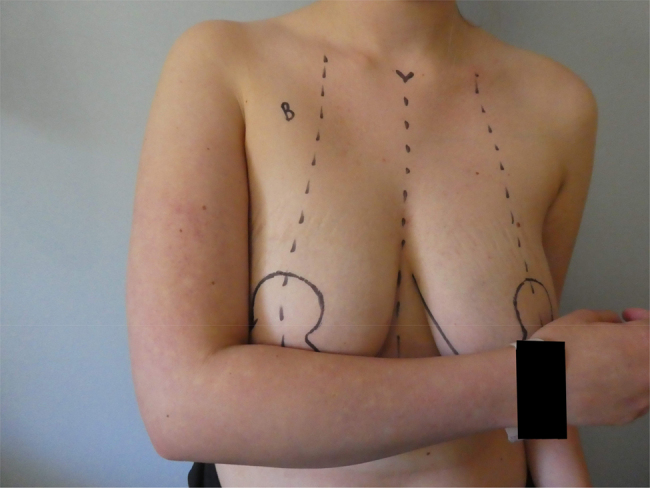
The new nipple areola complex position should sit within the arm and forearm with the elbow bent at 90°

Figure 1 shows a wise-pattern marking using the traditional method of transposing the level of inframammary fold onto the anterior breast skin and making this the position of the NAC.^1^ Our preference is to place the top of the NAC at this point because a nipple placed slightly low is much less of a concern than one placed too high. The new NAC position should sit between the arm and forearm when the elbow is flexed at 90° and placed across chest (Figure 2).

**Figure 3:**
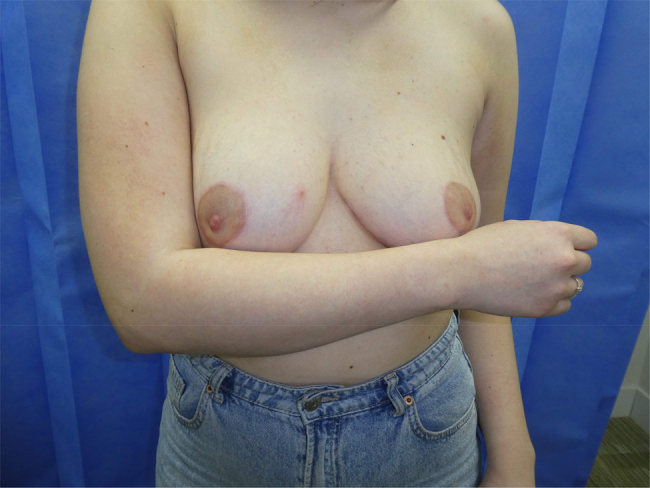
Post surgery with new nipple areola complex position sitting within the arm and forearm with the elbow bent at 90°

In addition, we have found that if one breast is significantly larger and with a lower NAC, the new NAC position is moved down slightly by approximately 1mm for every additional 4mm of breast ptosis on the larger side. This accounts for the increase in skin recoil that we have noticed above the new NAC when more weight is removed from the larger side. This can reduce postoperative NAC height difference in patients with breast asymmetry (Figure 3).

## Discussion

Although breast size and nipple position can vary significantly, upper limb length is usually identical. This technique has been found to be reliable in checking the new NAC has placed in the correct position in both BBR and mastopexy.
